# Draft genome sequence of *Moesziomyces globuligerus* strain NYNU178249, an asexual yeast-like smut fungus from rice leaves

**DOI:** 10.1128/mra.01404-25

**Published:** 2026-03-13

**Authors:** Chunyue Chai, Bingyan Song, Jiamin Song, Dan Lu

**Affiliations:** 1 School of Life Science, Nanyang Normal University 71072, Nanyang, People's Republic of China; 2 Research Center of Henan Provincial Agricultural Biomass Resource and Technology, Nanyang Normal University 71072, Nanyang, People's Republic of China; University of Strathclyde, Glasgow, United Kingdom

**Keywords:** smut fungi, genome assembly, asexual

## Abstract

We present the draft genome sequence of *Moesziomyces globuligerus* strain NYNU178249, an asexual yeast-like smut fungus from rice leaves in China. This long-read-based 18.6 Mb assembly provides a genomic resource for comparative studies of smut fungi, plant associations, and biotechnological potential.

## ANNOUNCEMENT

**Fig 1:**
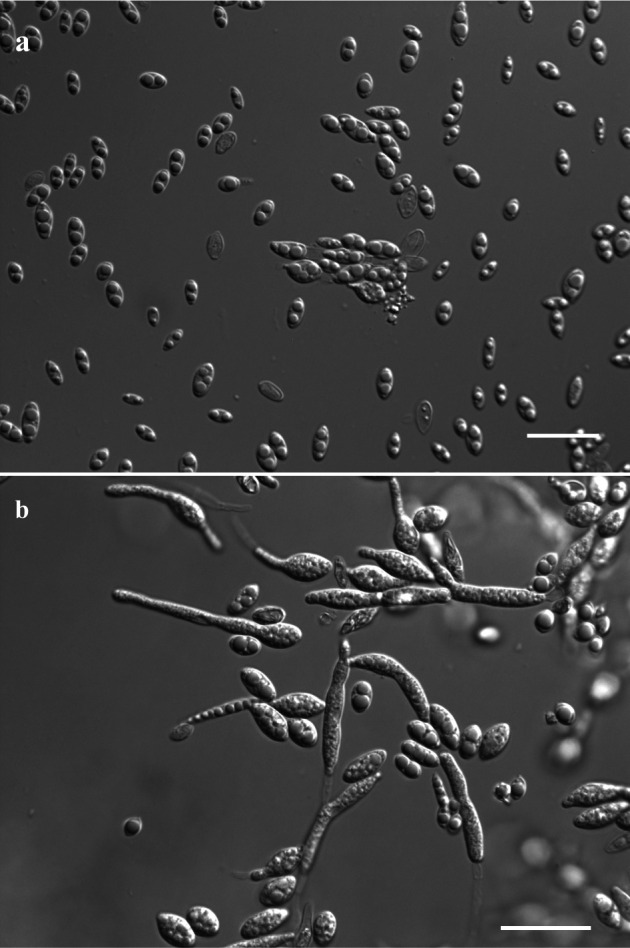
Yeast-like colony morphology and cell morphology of *Moesziomyces globuligerus* strain NYNU178249 grown on YPD agar. (**a**) Ungerminated conidia. (**b**) Germinated conidia. Scale bars: 20 µm.

*Moesziomyces* species are smut fungi that often display both yeast-like and filamentous plant-parasitic morphs and are noted for pathogenicity and biotechnological potential (1–3). However, genomic data for *Moesziomyces globuligerus* remain scarce (4). We sequenced the genome of isolate NYNU178249 to provide a foundational resource for comparative and functional studies. The isolate was sampled from symptomatic rice leaves collected in Baotianman National Nature Reserve, Henan Province, China (33.49°N, 111.94°E). Symptomatic leaf tissues were surface-sterilized (75% ethanol, 30 s; 1% NaClO, 1 min), rinsed with sterile water, cut into small pieces, and plated on yeast extract–peptone–dextrose (YPD) agar (Sangon Biotech, Shanghai, China). Emerging yeast-like colonies were purified by single-colony isolation. The isolate has been deposited in the China Center of Industrial Culture Collection (CICC 33281). Yeast-like colonies were cream to pale pink, with ovoid to ellipsoidal cells (Fig. 1). For genomic DNA preparation, the isolate was cultured in YPD broth at 25°C for 3 days with shaking (180 rpm) and harvested by centrifugation. Species identification was supported by morphology and by previously generated the internal transcribed spacer (ITS) region (MG255717; primers ITS1/ITS4) (5) and the large subunit (LSU) ribosomal DNA (MG255713; primers NL1/NL4) (6) sequences obtained by PCR and Sanger sequencing. The ITS sequence was queried using BLASTn in BLAST+ version 2.17.0 (NCBI; accessed 22 January 2026) against the NCBI nucleotide (nr/nt) database with default parameters; the top matches were *M. globuligerus* (MK027037) with 100% identity over 710 bp.

Genomic DNA was extracted using the Fungal Genomic DNA Extraction Kit (Tiangen Biotech, Beijing, China) according to the manufacturer’s instructions. High-molecular-weight DNA quality and quantity were assessed by 1% agarose gel electrophoresis. The genomic DNA was sheared to an average fragment size of approximately 15 kb using g-TUBE devices (Covaris, USA). A long-insert SMRTbell library was prepared using the SMRTbell Express Template Prep Kit 2.0 (Pacific Biosciences, Menlo Park, CA, USA) following the manufacturer’s protocol (7). The SMRTbell library was sequenced on a PacBio Sequel II platform (Pacific Biosciences) using Sequel II Binding Kit 2.0 and SMRT Cell 8M chemistry, yielding 464,816 raw reads with a total of 7.78 Gb of data and a read length *N*_50_ of 20,760 bp. Adapter sequences and low-quality subreads were removed using PacBio SMRT Link (version 13.1) with default filtering parameters. The resulting long reads were corrected and assembled with MECAT2 (version 2020.02.28) (8), and the draft assembly was polished with Racon (version 1.4.13) (9) and Pilon (version 1.22) (10) using default parameters.

The final assembly comprised 24 contigs with an *N*_50_ of 774,474 bp and a total size of 18,584,977 bp, with a GC content of 61.45%. PacBio long reads provided ~420× coverage of the 18.6 Mb genome. Genome completeness was evaluated with BUSCO (version 3.0.2) (11) using the Basidiomycota data set and indicated 738 complete genes (97.4%), supporting a near-complete assembly. The assembly offers a high-quality reference for comparative genomics within *Moesziomyces* and related Ustilaginomycetes and will facilitate studies of host association, genome evolution, and candidate genes underlying dimorphism and plant interaction. This genome provides a useful reference for investigating smut fungal evolution and plant-associated lifestyles.

## Data Availability

The Whole Genome Shotgun project for *Moesziomyces globuligerus* strain NYNU178249 has been deposited in DDBJ/ENA/GenBank under accession no. JARVLF000000000. The version described in this paper is the first version, JARVLF010000000. The associated BioProject and BioSample are available under accession nos. PRJNA954958 and SAMN34161017, respectively. Raw sequence reads have been deposited in the NCBI Sequence Read Archive (SRA) under accession no. SRR36284524.
